# Data driven contagion risk management in low-income countries using machine learning applications with COVID-19 in South Asia

**DOI:** 10.1038/s41598-023-30348-x

**Published:** 2023-03-06

**Authors:** Abu S. Shonchoy, Moogdho M. Mahzab, Towhid I. Mahmood, Manhal Ali

**Affiliations:** 1 Florida International University, 11200 SW 8th Street, Miami, FL 33199 USA; 2 Stanford University, 473 Via Ortega, Stanford, CA 94305 USA; 3 Texas Tech University, 2625 Memorial Circle, Lubbock, TX 79409 USA; 4 University of Leeds, Maurice Keyworth Building, Leeds, LS2 9JT UK

**Keywords:** Public health, Epidemiology, Health policy, Preventive medicine

## Abstract

In the absence of real-time surveillance data, it is difficult to derive an early warning system and potential outbreak locations with the existing epidemiological models, especially in resource-constrained countries. We proposed a contagion risk index (CR-Index)—based on publicly available national statistics—founded on communicable disease spreadability vectors. Utilizing the daily COVID-19 data (positive cases and deaths) from 2020 to 2022, we developed country-specific and sub-national CR-Index for South Asia (India, Pakistan, and Bangladesh) and identified potential infection hotspots—aiding policymakers with efficient mitigation planning. Across the study period, the week-by-week and fixed-effects regression estimates demonstrate a strong correlation between the proposed CR-Index and sub-national (district-level) COVID-19 statistics. We validated the CR-Index using machine learning methods by evaluating the out-of-sample predictive performance. Machine learning driven validation showed that the CR-Index can correctly predict districts with high incidents of COVID-19 cases and deaths more than 85% of the time. This proposed CR-Index is a simple, replicable, and easily interpretable tool that can help low-income countries prioritize resource mobilization to contain the disease spread and associated crisis management with global relevance and applicability. This index can also help to contain future pandemics (and epidemics) and manage their far-reaching adverse consequences.

## Introduction

Coronavirus Disease 2019 (COVID-19), a highly contagious respiratory borne infection, and its variants are still spreading rapidly and have caused more than 6.4 million global deaths. Although scientists have developed pharmaceutical treatments (medicine, therapeutics) and vaccines, it is still uncertain how effective these pharmaceutical measures are against the future variants of COVID-19, as evident from the recent astounding surge in global caseloads with the latest variant of concern—Omicron. The series of COVID-19 waves in the last two years have generated tremendous stress on the health care systems, prompting public health professionals to recommend universal vaccination and boosting.

This situation is more acute in Low and Middle-Income Countries (LMICs), where a major share of the unvaccinated population resides (about 19.87% of the low-income country population received only one dose of vaccine compared to the global full vaccination rate of 62%)^1^. In the face of widespread poverty, deficient safety-net measures, high self-employment, and a sizable informal economy, LMICs remained vulnerable imposing mobility restrictions that showed adverse consequences—leading to unemployment, poverty, and starvation^2^. Moreover, limited data capacity, insufficient health infrastructure, resource limitations, and inadequate COVID-19 testing ability made it further difficult for the LMIC governments to mitigate and manage the COVID-19 spread (for example, facilitating widespread testing and vaccinations), which demonstrate that conventional crisis management policies by LMICs are not equipped to tackle a pandemic of such scale.

Efforts to contain future pandemics (and epidemics) and manage their far-reaching adverse consequences require smart crisis management—employing early warning systems, efficient planning, and targeted interventions. To this end, we proposed a data-driven strategy to prioritize the allocation of limited resources of LMICs for gaining efficiency in managing current COVID-19 response by (1) deriving a composite index of contagion risk (CR) index, grounded on infectious disease spreadability vectors of COVID-19 suggested by epidemiologists and public health experts; (2) validating the proposed CR-Index with publicly available sub-national (district) level COVID-19 data for South Asia (Bangladesh, India, and Pakistan), employing regression and machine learning (ML) methods; and (3) identifying high and low-risk zones to prioritize resource mobilization (such as vaccination roll-out) to contain the disease spread and associated crisis management, with global relevance and applicability.

There have been limited country-specific studies generating composite indices to rank areas in terms of health vulnerabilities, but none focused on a contagion risk-based index, especially for a large region containing multiple low-income countries, such as South Asia, home to one-fifth of global population. In disaster management, risk is defined as the ability of the population to absorb and ultimately recover from the effects of being exposed to a hazardous event, given the level of vulnerability of the population and the resources they have to mitigate its effects. Therefore, our first main contribution is proposing a contagion risk index to aid policymakers in low-income countries to prioritize resource allocation, and devise effective mitigation and reconstruction strategies for affected populations. Our second contribution relates to examining the predictive accuracy and generalization capability of our proposed risk index, CR-Index, using tools of machine learning and cross-validation based on time-series data. Thus, our study relates to a small but burgeoning literature that uses machine learning and artificial intelligence to predict COVID-19 outbreak^3–6^.

## Methods

### Data

The data employed in this study are collected from institutions and national statistics departments of respective countries. For Pakistan, we only use Sindh province in our estimations, due to the lack of district-level daily COVID-19 data. Similarly, for Bangladesh, we could not use COVID-19 mortality estimates. For each of the countries, we use different time-spans due to data availability issues. For Bangladesh, we use data from April 13, 2020, to February 28, 2022, for India, from January 30, 2020, to October 31, 2021, and for Pakistan (Sindh) from September 12, 2020, to November 22, 2021. Detailed data sources are provided in Table 1. Further details on the data collection approach and summary statistics are available in the supplementary file.

**Table 1:** Data sources.

Variables	Source of data
Bangladesh	
Population density	Bangladesh Population Census 2011, Bangladesh Bureau of Statistics
Informal employment	Quarterly Labor Force Survey 2016–2017, Bangladesh Bureau of Statistics
International migration	Household Income and Expenditure Survey 2016–2017, Bangladesh Bureau of Statistics
Internal migration	
Beds per million population	Directorate General of Health Services, Ministry of Health, Bangladesh
COVID-19 cases	Institute of Epidemiology, Disease Control and Research, Bangladesh
India	
Number of beds	Development Data Lab
Urban population	
In-migration	
Non-farm employment	
COVID-19 Cases	
COVID-19 Deaths	
Pakistan (Sindh)	
Intra migration	Pakistan Social and Living Standard Measurement 2019–2020
Urban population	Population Census 2017, Pakistan Bureau of Statistics
Health facilities	Pakistan Demographic and Health Survey
Informal employment	Labor Force Statistics 2017–2018
COVID-19 cases	Health Department, Government of Sindh
COVID-19 deaths	

### Domain and variable choice for CR-index

To derive the sub-national CR-Index, we broadly defined the following four domains founded on infectious disease spreadability vectors: *urbanization, informality, migration,* and *health infrastructure*.

Under the urbanization domain, we use district-wise percentage of the urban population variable for all the three countries^7–12^. This degree of urbanization variable (share of the urban population) captures a particular district's overall economic-demographic condition, demonstrating more vibrant economic interactions in the region. It also suggests a higher level of industrialization, trade, and travel connectivity resulting in a higher probability of social interactions and a greater possibility of spreading the virus.

The informal employment or “informality” at the sub-national level also plays a role in COVID-19 transmission, especially in LMICs. Informal employment does not follow conventional labor rights and policy. Moreover, employees hired informally are low-income daily wage earners who do not have job security, do not get paid or seek leaves, and often do not have the luxury to work from home, resulting in more exposure to contagion spread. Developing countries, such as Bangladesh, India, and Pakistan have a considerably large informal sector (both in rural and urban areas), hired in construction, transportation, manufacturing, and service sectors, where the continuous search for new income opportunities is a harsh reality. Approximately 71% of the sectoral composition of employment in developing countries are self-employed compared to 13% in developed countries^13,14^. We obtained data on informal employment share for Bangladesh and Pakistan (Sindh), but such data with high accuracy was not available for India, where we used the share of non-farm employment in India, which is a reasonable proxy for informal employment^15–17^.

Developing countries are also known to have a large share of the mobile population—classified as domestic and international migrants. For example, Bangladesh is the sixth leading country of origin for international migrants^18^. Due to the COVID-19 pandemic and resulting global economic shutdown, a large percentage of international migrants went back to their home countries—carrying the disease with them and contributing to the virus spread. Similarly, domestic migrants face similar consequences and lost urban sources of income due to the lockdown that forced them to go back to their villages of origin. Both these channels of reverse migration also contributed to virus transmission, as documented in recent studies^19,20^. To capture this important vector of virus diffusion, we include district level international and domestic migrants as a variable in the CR-Index construction^19–21^. For Bangladesh and India, we obtained data on the share of migrants in the district that includes both domestic and international migrants. For Pakistan, only the data on domestic migration at the district level was available.

Finally, sub-national health infrastructure, measured as the number of beds and health facilities per million of population, have been utilized as candidate variables representing the health infrastructure domain^22–24^. For Bangladesh and India, we used hospital beds per million in a district. For Pakistan, we took health facilities per million as data were more reliable for health facilities than the total number of beds.

We tried to be consistent about the choice of variables for the four domains across the three countries. However, due to administrative and institutional differences (along with data availability), a few variables are not the same. Sources for data for each of the domain specific variables for each of the countries are provided in Table 1.

The CR-Index variable is a composite of these four domains, whereby greater its value, greater the risk a district faces in terms of spread of COVID-19 infections to its population. Further details on the data used for the CR-Index, its construction, rationale, and descriptive statistics are given in subsections A1, A2, and A3 in the supplementary file.

### CR-index construction

Using each of the domain specific variables, we first created sub-indices *j* {*j* = 1, 2, 3, 4} using the following formula for each district* d* in country *c* {*c* = *Bangladesh*, *India*, *Pakistan*}:

1 $$ SubIndex_{c,d,j} = \frac{{\left( {X_{c,d,j} - \min \left( {X_{c,d,j} } \right) } \right)}}{{\left( {\max \left( {X_{c,d,j} } \right) - \min \left( {X_{c,d,j} } \right) } \right)}} $$

${X}_{c,d,j}$ is the sub-national domain specific value*,* while $\mathrm{min}\left({X}_{c,d,j}\right)$ and $\mathrm{max}\left({X}_{c,d,j}\right)$ are the country-domain specific minimum and maximum values of the variable at the district (sub-national) level. After generating sub-indices for each domain-district-country, we use simple arithmetic mean to generate the composite CR-Index for each district in each country, following the human development index (HDI) construction method of the UNDP for wide acceptability^25^. Formally,

2 $$ CRIndex_{c,d} = \frac{{\mathop \sum \nolimits_{j = 1}^{n} SubIndex_{c,d,j} }}{n} $$

Here *n* is the number of domains. We used arithmetic mean, given some sub-indexes generated zero value (for example, some districts did not have any reported international migration, resulting in a score of zero for that sub-index). The process of CR-Index construction normalizes the variables based on the minimum and maximum variation of each variable across sub-nations for each country.

### Measures for CR-index validation

#### Correlation test

We run an Ordinary Least Squares (OLS) regressions to examine whether the statistical validity of the correlation between the CR-Index and COVID-19 epidemiologic data remains consistent across the time-horizon after controlling for district-specific variables. We run the following regressions:

3 $$ IHST \left[ {y_{c,d,t} } \right] = \alpha + \beta \cdot CRIndex_{c,d} + \gamma \cdot Z_{c,d} + \epsilon_{c,d,t} $$

Here, *t* represents a specific time horizon (week) where $t=\mathrm{1,2},\dots , n$ and $IHST[{y}_{c,d,t}]$ is the Inverse Hyperbolic Sine Transformation (IHST) of the reported COVID-19 epidemiologic data (positive cases or death) for each district. IHST is appropriate when a variable contains a significant number of 0’s. The equation for IHST for a variable *y* is, log(*y*_*i*_ + (*y*_*i*_^2^ + 1)^0.5^) where, $y_{i} \ge 0$. The IHS ensures that zero COVID-19 cases or deaths are not dropped during the process of normalization. ${Z}_{c,d}$ is the district-specific control, which is the district level population. $\epsilon_{c,d,t}$ is a time and district specific unobservable term.

#### Regression test

Utilizing the time-series pattern of the daily data, we estimate regressions in Eqs. (4) and (6), where daily reported COVID-19 cases and deaths are regressed against the CR-Index using OLS. In Eqs. (5) and (7), the daily reported COVID-19 cases and deaths are regressed against the CR-Index, with day fixed effects (FE) and district controls. Under this setting, Eqs. (5) and (7) are the most conservative specifications, taking care of the time dimension and regional control, while Eqs. (4) and (6) control for the time trend, $\gamma \left(t\right)$.

4 $$ IHST\left( {Cases_{c,d,t} } \right) = \alpha + \beta \cdot CRIndex_{c,d} + \gamma \cdot t + \epsilon_{c,d,t} $$

5 $$ IHST \left( {Cases_{c,d,t} } \right) = \alpha + \beta \cdot CRIndex_{c,d} + \gamma \cdot Day_{t} + \Omega \cdot Population_{c,d} + \epsilon_{c,d,t} $$

6 $$ IHST\left( {Deaths_{c,d,t} } \right) = \alpha + \beta \cdot CRIndex_{c,d} + \gamma \cdot t + \epsilon_{c,d,t} $$

7 $$IHST\left( {Deaths_{c,d,t} } \right) = \alpha + \beta \cdot CRIndex_{c,d} + \gamma \cdot Day_{t} + \Omega \cdot Population_{c,d} + \epsilon_{c,d,t}$$

In Eqs. (4) and (6), the time trend, $\gamma \left(t\right)$, controls for the rise of district specific COVID-19 cases throughout the study period. The parameter $\gamma$ in Eqs. (5) and (7) is the time FE, which controls for any time related variation across days within the study period for all the districts. $\epsilon_{c,d,t}$, is the time and district specific unobservable term.

#### Risk zoning and predictive accuracy using machine learning

To cross-validate the predictive accuracy of the CR-Index for any future event, we use the Random Forests (RF) process in machine learning^26,27^. RF belongs to a class of supervised algorithms and has been used widely in public health and epidemiology^28,29^. In contrast to non-parametric approaches, RF allows for flexible functional forms leading to better predictions and has been proven to effectively manage the bias-variance trade-off^27^.

Based on CR-Index score, we classified districts into three risk zones: ‘Red’ if the percentage of COVID-19 cases (or deaths) exceeds the 90th percentile relative to all other districts for a given country; ‘Orange’ if the percentage of cases/deaths is between 90 and 25th percentiles; and ‘Green’ if below the 25th percentile. Using the RF algorithm, the multiclass classification response is modeled as a function of CR-Index, where, for a given country, the predicted risk zone of each district is compared with their actual risk in the test data. We use the early/late split method^30^, where the early data is utilized to train, and the later data is used as a test^31^. The results of the random forests and predictive accuracy are determined by two key parameters also known as tuning parameters: the number of trees to grow and the number of random features for the splitting process^32^. The number of trees is set to 500, and the number of random features is one, since our primary variable of interest is the CR-Index. Smaller the number of features, greater the parsimony of the model, therefore, greater the predictive accuracy^27,32^. Moreover, to reduce the bias and variance of the estimations, we fit RF using fivefold cross validation repeated for 50 times^32^.

Finally, the classification prediction of the CR-Index is assessed by the AUC (Area Under the Curve) and ROC (Receiver Operating Characteristics) curves. ROC is a probability curve, whereas AUC measures the degree of separability^33^. The ROC curve plots the true positive rate (TPR) against the false positive rate (FPR). TPR, also known as “Sensitivity”, is the ratio of districts that are correctly categorized as high-risk (true positive) to the total number of positives. Whereas FPR, is “1—Specificity)”, where “Specificity” is the true negative rate, and it is the ratio of the number of low-risk districts incorrectly categorized as high-risk to the total number of actual negatives. In general, an AUC value between 0.70 and 0.80 is considered acceptable; 0.80–0.90 is considered excellent, and more than 0.90 is considered outstanding^33^. The values of AUC-ROC are obtained using the “mLeval’’ and “pROC” packages of statistical software “R”^34,35^.

### Ethics approval

Prior ethical approval is not required as the study uses publicly available data.

## Results

### Correlation estimates

**Figure 1:**
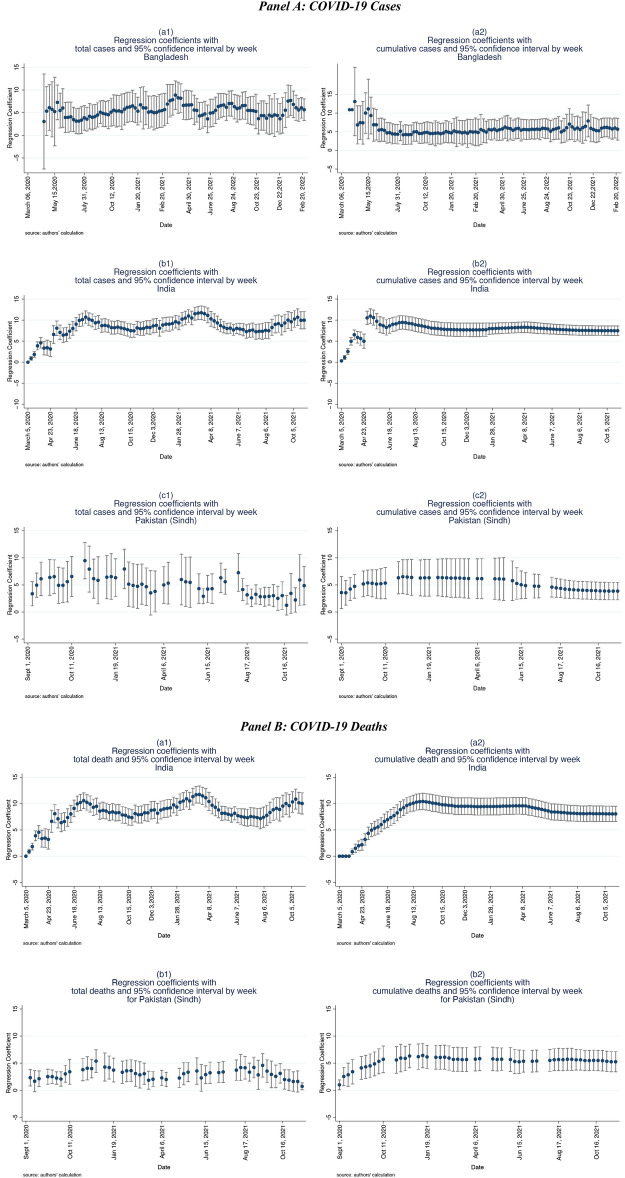
Weekly OLS regression estimates.

Based on Eq. (3), Panel A of Fig. 1, produced the weekly regression estimates for the CR-Index for district-wise COVID-19 cases for Bangladesh, India, and Pakistan (Sindh). During the initial stages of the pandemic, few of the estimates came out statistically insignificant due to insufficient data-points across all the districts. However, after COVID-19 cases were reported from almost all the districts, estimates remained robust and statistically significant. Panel B presents the weekly regression estimates using COVID-19 deaths. Both the figures show that CR-Index is statistically significant and maintains a close range of coefficient values within the period of our analysis.

### Panel regression estimations

Columns (1) and (3) in Table 2 report the regression results based on Eqs. (4) and (6) for COVID-19 cases using OLS and time FE. Whereas columns (2) and (4) report the regression results based on Eqs. (5) and (6) for COVID-19 deaths. In summary, the OLS and FE regression results show that the CR-Index is positive and significantly associated with COVID-19 spread and mortality, demonstrating strong predictive power of the CR-Index. In Column (2), 1 percent increase in the value of CR-Index is associated with an approximate 1.88 percent increase in the number of positive COVID-19 cases for Bangladesh, 5.39 percent for India, and 4.6 percent for Pakistan (Sindh), after controlling for time fixed effect and district specific control. In Column (4), a 1 percent increase in CR-Index is associated with an approximate 1.65 and 1.06 percent increase in COVID-19 deaths for India and Pakistan (Sindh). Columns (2) and (4) offer the most conservative specification, and with regular assumptions on FE identification. The *p* values for these coefficients are close to zero, showing a strong predictive power of the CR-Index in case of COVID-19 related deaths as well.

**Table 2:** OLS and FE regression models.

Variables	Country	(1)	(2)	(3)	(4)
		OLS	FE	OLS	FE
		IHS (Daily cases)	IHS (Daily cases)	IHS (Daily death)	IHS (Daily deaths)
CR index	Bangladesh	5.552***	1.884***		
		(0.067)	(0.053)		
Observations		36,577	36,577		
R-squared		0.50	0.67		
CR-index	India	7.576***	5.389***	2.447***	1.651***
		(0.029)	(0.029)	(0.015)	(0.014)
Observations		375,298	375,298	375,381	375,381
R-squared		0.48	0.54	0.27	0.33
CR index	Pakistan (Sindh)	4.802***	4.604***	1.450***	1.058***
		(0.065)	(0.086)	(0.036)	(0.029)
Observations		10,314	10,314	10,314	10,314
R-squared		0.46	0.45	0.32	0.33
Time fixed effect (Daily)		No	Yes	No	Yes
Time trend		Yes	No	Yes	No
District specific control		No	Yes	No	Yes

Robust standard errors in parentheses. *** *p* < 0.01, ** *p* < 0.05, * *p* < 0.1.

### Contagion zoning

**Figure 2:**
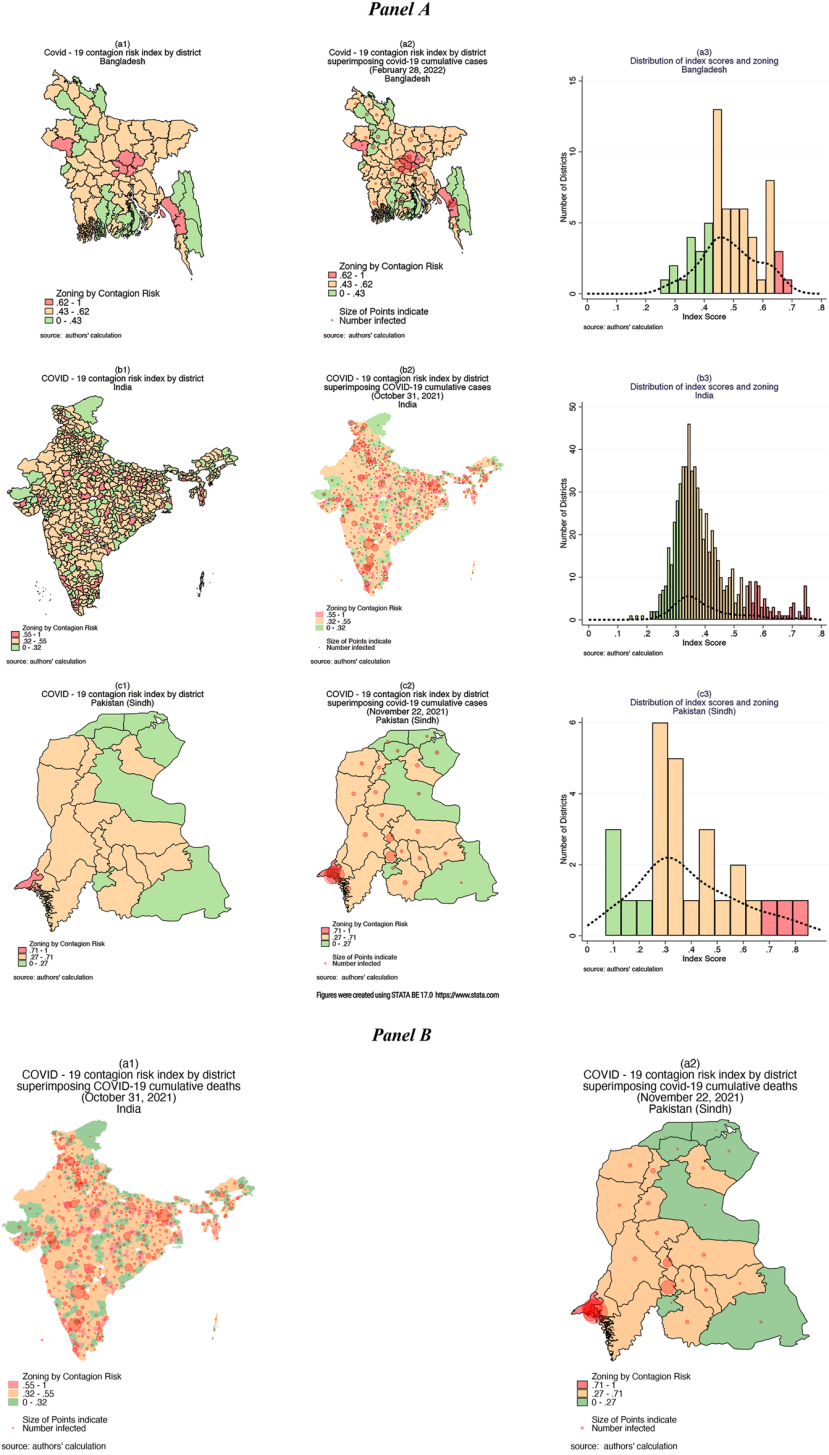
Contagion-risk zoning maps.

Using the score of the CR-Index, we divided districts for each of the countries into three zones (Red/Orange/Green) based on the CR-Index distribution described before. In Fig. 2, Panel A, sub-panels (A1), (B1), and (C1) show these three zones’ spatial distribution for each of the countries. Sub-panels (A2), (B2), and (C2) superimpose the latest available aggregated district-wise COVID-19 positive cases. The map shows that the proposed index and zoning classification can identify vulnerable districts/regions robustly. Sub-panels (A3), (B3) and (C3) show the CR-Index histogram and kernel-density plot of the distribution. Panel B of Fig. 2 superimposes the aggregate COVID-19 deaths for India and Pakistan (Sindh) on their respective maps and shows that the CR-Index is robustly identifying vulnerable regions with high COVID-19 death rates. All the maps in Fig. 2 were generated using the statistical software, STATA BE 17 (https://www.stata.com/). Section C (supplementary file) reports the basic summary statistics, and the distribution of district-specific CR-Index scores COVID-19 cases and deaths (as a % of total reported cases for the latest available date) based on these proposed zones.

### Machine learning results

Table 3 reports the AUC values from the machine learning exercise for the three South Asian countries. Columns (1)–(5) of Table 3 report estimates using the entire 2020 as the training data and 2021 as the testing data—using the early-late split method of cross validation—our preferred model for the validation exercise. Using the same training data we also predicted the month-by-month results, where each month in 2021 was used as the test, and reported the average of these in columns (6)–(10). Time series plots of COVID-19 cases and deaths for the three South Asian countries are provided in Section B of the supplementary file. The complete month-by-month results are given in Section D in the supplementary file.

**Table 3:** Predictive Performance of the CR Index using 2021 as test data and 2020 as training data (District wise).

	Test data: Year 2021 (Training data: 2020)					Test data: Each available months in 2021 (mean) (Training data: 2020)				
	India		Pakistan (Sindh)		Bangladesh	India		Pakistan (Sindh)		Bangladesh
	Cases	Death	Cases	Death	Cases	Cases	Death	Cases	Death	Cases
	(1)	(2)	(3)	(4)	(5)	(6)	(7)	(8)	(9)	(10)
AUC-ROC	0.90	0.84	0.97	0.96	0.86	0.73	0.77	0.87	0.84	0.76
Specificity	0.89	0.89	0.85	0.91	0.83	0.75	0.86	0.78	0.83	0.77
Sensitivity	0.83	0.83	0.83	0.90	0.74	0.61	0.56	0.69	0.70	0.62
Balanced accuracy	0.86	0.86	0.84	0.91	0.78	0.68	0.71	0.73	0.77	0.69

The table provides the predictive accuracy results using multiclass classification. Districts belong to the red zone if covid-19 cases are greater than or equal to the 90th percentile; orange zone if cases fall within the 90th and 25th percentile; and green zone if below the 25th percentile. Column (1)–(5) use the entire 2020 as the training data and 2021 as the testing data using the early-late split method of cross validation. Column (6)–(10) report the means computed using the 12-months average across all the available months in 2021 used separately as testing data and 2020 as the training data.

From columns (1)–(5) in Table 3, the mean values of AUCs range from 0.84 to 0.97, therefore, suggesting excellent to outstanding out-of-sample predictive accuracy for the CR-Index. This implies that for a given random data, the CR-Index can distinguish districts with high-risk COVID-19 cases/deaths for more than 91% of the time, on average. These findings are further supported by results in columns (6)–(10) of Table 3 using month-by-month out-of-sample prediction tests, where the average value of AUC is about 0.80. In addition to AUC-ROC, Table 3 also reports the values of other commonly used metrics for predictive performance, namely, sensitivity, specificity, and balanced accuracy.

To check the robustness of the multiclass classification based estimates, we additionally conduct binary risk-zoning classification (high-risk vs. low-risk districts using 75th percentile cut-off) and district-monthly data. These estimates are strongly similar with Table 3 estimates and are reported in the supplementary file (section D2 and D3). The ROC curves corresponding to binary classification are also reported in Fig. D1 (supplementary file).

## Discussion

Data-driven mitigation planning, and management are crucial for resource-constrained countries—ensuring the best use of their limited resources. In the absence of adequate, regular and reliable epidemiologic data, it is difficult for the policy makers from low-income countries to derive advance planning and timely response to contain and manage the spread of an epidemic or pandemic; and formulate a targeted resource distribution plan for mass testing, therapeutics and vaccination.

To this end, this paper created a sub-national level contagion-risk index in South Asia, developed using four important socio-economic and health domains (urbanization, informality, migration and health infrastructure) on disease spreadability vectors. The dataset employed for CR-Index utilized readily available statistics at the sub-national level, which has wide applicability for global adoption, particularly for LMICs. Our proposed CR-Index shows strong statistical properties in explaining the variabilities of COVID-19 cases and deaths for all the three countries, across different timeframes. We have provided a simple flowchart, Fig. A5.1, Section A5, in the supplementary file to explain the step-by-step process to develop the CR-Index for any LMICs where such data are available.

Built on the CR-Index, we propose zone-specific contagion management measures as a potential solution for precautionary advance planning and to prioritize the efficient allocation of limited resources. Classifying districts into high, moderate and low-risk, based on the 75th and 25th percentile distribution thresholds, we validate the CR-Index accuracy using machine learning based on test/train cycle method^26^. As suggested in the existing literature to use a nonlinear curve fitting strategy^36^, we utilize random forest as our machine learning algorithm, which allows us to explore non-linearities in our proposed CR-Index variable. We applied 2020 COVID-19 data to train the CR-Index, and then examine its out-of-sample predictive performance using the data in 2021—the test sample. The resultant values of AUC-ROC in our preferred model are generally well-above 0.80 and statistically significant at a 5% significance-level, thus, suggesting superior predictive performance of the CR-Index. The predictive performance of the index is more accurate during periods of exceptionally high COVID-19 cases and deaths. For example, the values of AUC-ROC using the data for COVID-19 cases in India for the months of April and May 2021 (when delta was the dominant variant in India) as test samples are the highest amongst all the months in 2021 (supplementary Table D1.1).

The findings of this study relate to recent studies that have computed composite indices to rank districts in terms of COVID-19 vulnerabilities using various information on socio-economic, demographic, and infrastructure characteristics^7,10,11,37–39^. However, our study differs from those studies in some important ways. First, instead of creating COVID-19 vulnerability indices, our study focuses on computing contagion risk indexes at sub-national level. Second, while these studies focus on a single country, for example, India^3^, our study, in contrast, computes contagion risk indices for a region, using data from major three countries in South Asia with a combined population of more than 1.5 billion (about one-fifth of global population). Third, there exists paucity of studies that examine the validity of the relevant vulnerability indices for COVID-19 at sub-national level. Our study aims to fill this void by using machine learning where the performance of the contagion risk index is validated using various hold-out data.

Despite the strong empirical and predictive support of the CR-Index, there are some important limitations. First, it would have been ideal to construct a measure of the index at a lower administrative level, such as at the level of a sub-district. Second, our measure of CR-Index is static, and the data utilized to construct the index is not the most recent, therefore, it may not accurately capture riskiness in districts in which rapid changes have transpired lately. Future research can employ more recent and real-time data that are collected from disparate sources, such as, phone-tracking data to capture mobility and migration. Third, the rationale for choosing variables under each domain is country-specific, and this choice could be arbitrary. However, the focus should not be on selecting variables but on the domains, which are known to affect the contagion spread during this pandemic. Fourth, we have constructed the CR-Index using variables for each country and normalized the variables based on the minimum and maximum variation of each variable across districts. However, relative values of the variables can also be utilized to develop the index. Fifth, our index assigns equal weights to each variable and sub-domain. The domains may not be equally important; thus, different weights could be assigned to the variables. Alternatives, such as, factor weights are, however, problematic as they are sensitive to data and lack interpretability. Sixth, due to inaccuracy and non-availability of data on COVID-19 induced deaths at the district-level of Bangladesh, we could not use it for the CR index construction for Bangladesh. Finally, our proposed CR-Index is simple and additive, and, therefore, unable to account for the multiplicative and non-linear nature of the variables and sub-domains. This requires the use of methodological advancements using non-linear/non-parametric methods that can create a more robust measure of the contagion risk index.

Despite reduction in COVID-19 case trends and ever-increasing vaccination rate, epidemiologists and virologists expressed vigilance and have not completely ruled out future variants that can evade vaccine immunity^40^. The proposed contagion risk index aims to help policymakers in LMICs to effectively prioritize resource allocation and adopt risk mitigation strategies for better responses to future COVID-19 waves and pandemics. The analysis in this paper offers initial guidelines for developing a comprehensive contagion risk index, which can be modified further with additional data and methodological enhancements.

## Conclusion

In conclusion, in the absence of real time surveillance data, our study proposes a system for early warning and potential contagion hotspots—based on communicable disease spreadability vectors—for better disease management, planning and targeted intervention. To facilitate this recommendation, we have developed, validated, and illustrated the use of an easily interpretable data-driven Contagion Risk Index, as a toolkit, in the case of COVID-19 pandemic in South Asia. This simple, low-cost, flexible toolkit could be readily adapted for low and middle-income countries and could be utilized as a decision support guide for an efficient use of their limited resources, while reducing the likelihood of a rapid resurgence of the communicable diseases which would be useful for future epidemics and pandemics.

## Supplementary Information

Supplementary Information.

## Data Availability

Data and codes to reproduce the analysis and graphs associated with this study are available from https://github.com/ma0511/COVID-19-South-Asia-Project.
