# Supplementary material for: Data driven contagion risk management in low-income countries using machine learning applications with COVID-19 in South Asia
Source: Sci Rep. 2023 Mar 6;13:3732. doi: 10.1038/s41598-023-30348-x (PMC9987367; doi:10.1038/s41598-023-30348-x)

# **A. Data & Variable Description for CR-Index**

## **A1: Data and Variable Selection**

All the data sources used in this study are listed in the sub-section below. For Pakistan, national level district-wise disaggregated data was not available, except for the Sindh province. However, among the 29 districts of the province, we were able to collect information from 27 districts. Similarly, Bangladesh did not report district-level daily COVID-19 mortality estimates.

It is important to note that we have considered a total of 19 potential variables under the four domains. However, we have included only the variables with strong statistical and economics association with daily COVID-cases of Bangladesh, India and Pakistan, respectively for the index construction.

We have district-wise CoVID-19 deaths along with COVID-19 cases as the outcome variable for verifying CR-Index’s validity in India. The data on COVID-19 cases and deaths for India are available in the form of cumulative numbers for each day. As our goal is to evaluate the strength of the CR-Index, it is crucial to get daily COVID-19 data. For that, we have taken the first difference of daily cumulative data for India on COVID-19 cases/deaths to get the number of COVID-19 positive cases and reported deaths. We followed the following equation:

$$Cases/Deaths_{t}=Cumulative Cases/Deaths_{t}- Cumulative Cases/Deaths_{(t-1)}$$

This method faced a problem. When we take the first difference of the cumulative cases, we get a few observations with negative values. Our assumption is that these are enumeration errors and hence we replaced these values with zeros. For both positive cases and deaths, merely 1% of the total sample faced this issue in India.

**A2: Variable Description, Domain and Rational**

Table A2.1: CR-Index Variables

| **Domain** | **Rational** | **Citation** | **Country** | **Variable (district level)** |
| --- | --- | --- | --- | --- |
| Urbanization | Captures a particular district's overall economic activities, industrialization, trade, and travel connectivity | [7-12] | Bangladesh | Share of urban population |
|  |  |  | India |  |
|  |  |  | Pakistan |  |
| Informality | Informal employment is not associated with paid leave or seek leave and do not allow work from home facilities, results in more exposure to COVID-19 | [15-17] | Bangladesh | Share of informal employment in the district |
|  |  |  | India | Share of non-farm employment |
|  |  |  | Pakistan | Share of informal employment in the district |
| Migration | Migration is an important channel for COVID-19 spread | [19-21] | Bangladesh | Share of out migration |
|  |  |  | India |  |
|  |  |  | Pakistan | Share of in migration |
| Health | Health infrastructure to support COVID-19 patients | [22-24] | Bangladesh | Number of public hospital beds available per million population |
|  |  |  | India |  |
|  |  |  | Pakistan | Number of public health facilities available per million population |

We obtained data on informal employment share for Bangladesh and Pakistan (Sindh), but such data with high accuracy was not available for India, where we used the share of non-farm employment in India, which is a reasonable proxy for informal employment. We could not get access to out-migration data for Sindh from the source (Pakistan Social and Living Standard Measurement 2019-20).

**A3: Summary Statistics**

Table A3.1: Summary Statistics (Bangladesh)

| Variable | Obs | Mean | Std. dev. | Min | Max |
| --- | --- | --- | --- | --- | --- |
| Daily COVID-19 Cases (Until February 2022) | 36,577 | 44.85 | 210.64 | 1.00 | 6526.00 |
| Share of out migration (International & Internal) | 36,577 | 0.13 | 0.10 | 0.01 | 0.41 |
| Share of informal employment | 36,577 | 0.84 | 0.08 | 0.53 | 0.97 |
| Share of urban population | 36,577 | 0.31 | 0.22 | 0.11 | 0.89 |
| Beds per million population | 36,577 | 223.56 | 126.15 | 17.89 | 747.88 |

Table A3.2: Summary Statistics (India)

| Variable | Obs | Mean | Std. dev. | Min | Max |
| --- | --- | --- | --- | --- | --- |
| Daily COVID-19 Cases (Until October 2021) | 375,299 | 199.76 | 3054.65 | 0.00 | 135293.00 |
| Daily COVID-19 Deaths (Until October 2021) | 375,381 | 1.11 | 6.92 | 0.00 | 1074.00 |
| Beds per million population | 420,488 | 629.17 | 743.39 | 0.00 | 6246.88 |
| Share of urban population | 420,488 | 0.26 | 0.21 | 0.00 | 1.00 |
| Share of out migration (International & Internal) | 420,488 | 0.00 | 0.00 | 0.00 | 0.05 |
| Share of Non-farm Employment | 420,488 | 0.46 | 0.21 | 0.11 | 1.00 |

Table A3.3: Summary Statistics (Pakistan-Sindh)

| Variable | Obs | Mean | Std. dev. | Min | Max | |
| --- | --- | --- | --- | --- | --- | --- |
| Daily COVID-19 Cases (Until November 2021) | 10,314 | 24.06 | 56.61 | 0.00 | | 958.00 |
| Daily COVID-19 Deaths (Until November 2021) | 10,314 | 0.34 | 1.19 | 0.00 | 21.00 | |
| Health Facilities per million population | 10,314 | 0.51 | 0.27 | 0.00 | 1.00 | |
| Share of In-migration | 10,314 | 2.99 | 2.72 | 0.16 | 10.76 | |
| Share of urban population | 10,314 | 41.24 | 27.62 | 8.05 | 100.00 | |
| Share of informal employment | 10,314 | 0.42 | 0.17 | 0.17 | 0.78 | |

**A4: Index Score**

*District Index scores by zone and % share of actual cases by the district*

Table A4.1 Index Scores (Bangladesh)

*% of actual cases drawn from February 10, 2022*

| **Red Zone** | **CR-Index** | **% Of Actual Cases** | **Green Zone** | **CR-Index** | **% Of Actual Cases** |
| --- | --- | --- | --- | --- | --- |
| Chittagong | 0.69 | 6.27 | Mymensingh | 0.56 | 1.3 |
| Dhaka | 0.66 | 32.49 | Madaripur | 0.54 | 1.25 |
| Gazipur | 0.65 | 2.07 | Maulvibazar | 0.53 | 0.75 |
| Narsingdi | 0.65 | 0.97 | Shariatpur | 0.53 | 0.59 |
| Narayanganj | 0.63 | 1.65 | Faridpur | 0.53 | 2.15 |
| Rajshahi | 0.62 | 1.28 | Thakurgaon | 0.53 | 0.2 |
| Munshiganj | 0.62 | 1.19 | Khulna | 0.52 | 0.97 |
| Manikganj | 0.61 | 0.86 | Kushtia | 0.52 | 1.03 |
| Comilla | 0.61 | 3.89 | Cox's Bazar | 0.51 | 4.57 |
| Brahmanbaria | 0.61 | 1.76 | Jessore | 0.51 | 1.23 |
| Chandpur | 0.61 | 1.3 | Gaibandah | 0.51 | 0.26 |
| Tangail | 0.61 | 1.85 | Kishoreganj | 0.51 | 1.49 |
| Feni | 0.6 | 1.23 | Sherpur | 0.5 | 0.18 |
| Jhenaidah | 0.57 | 0.4 | Sunamganj | 0.49 | 1.19 |
| Noakhali | 0.56 | 1.8 | Barguna | 0.48 | 0.15 |
| Rajbari | 0.56 | 0.88 | Barisal | 0.48 | 1.28 |
|  |  |  | Lakshmipur | 0.47 | 1.06 |
|  |  |  | Jamalpur | 0.47 | 0.26 |
|  |  |  | Nilphamari | 0.46 | 0.29 |
|  |  |  | Netrokona | 0.46 | 0.31 |
|  |  |  | Rangpur | 0.46 | 1.12 |
|  |  |  | Pabna | 0.46 | 2.26 |
|  |  |  | Magura | 0.45 | 0.26 |
|  |  |  | Naogaon | 0.45 | 0.75 |
|  |  |  | Chuadanga | 0.44 | 0.59 |
|  |  |  | Habiganj | 0.44 | 1.08 |
|  |  |  | Satkhira | 0.44 | 0.73 |
|  |  |  | Sylhet | 0.44 | 1.96 |
|  |  |  | Sirajganj | 0.44 | 0.99 |
|  |  |  | Chapai Nababganj | 0.44 | 0.29 |
|  |  |  | Panchagarh | 0.43 | 0.48 |
|  |  |  | Narail | 0.43 | 0.68 |
|  |  |  | Bogra | 0.43 | 0.97 |
|  |  |  | Pirojpur | 0.42 | 0.15 |
|  |  |  | Lalmonirhat | 0.42 | 0.44 |
|  |  |  | Kurigram | 0.41 | 0.22 |
|  |  |  | Natore | 0.4 | 0.59 |
|  |  |  | Rangamati | 0.39 | 0.15 |
|  |  |  | Patuakhali | 0.38 | 0.59 |
|  |  |  | Jhalokati | 0.38 | 0.15 |
|  |  |  | Dinajpur | 0.36 | 0.99 |
|  |  |  | Joypurhat | 0.36 | 0.4 |
|  |  |  | Gopalganj | 0.35 | 1.65 |
|  |  |  | Khagrachhari | 0.35 | 0.15 |
|  |  |  | Bhola | 0.32 | 0.64 |
|  |  |  | Meherpur | 0.3 | 0.95 |
|  |  |  | Bagerhat | 0.28 | 0.37 |
|  |  |  | Bandarban | 0.27 | 0.02 |
| **Total** |  | **59.86** |  |  | **40.13** |

Table A4.2: Index Scores (India)

*% of actual cases drawn from October 31, 2021*

| **Red Zone** | **CR-Index** | **% of Actual Cases** | **% of Actual Deaths** | **Green Zone** | **CR-Index** | **% of Actual Cases** | **% of Actual Deaths** |
| --- | --- | --- | --- | --- | --- | --- | --- |
| Kollam | 0.76 | 0.12 | 2.46 | Krishnagiri | 0.44 | 0.02 | 0.41 |
| Mungeli | 0.75 | 0.13 | 1.23 | Jogulamba | 0.44 | 0 | 0 |
| Chatra | 0.75 | 0.05 | 1.64 | Umaria | 0.44 | 0 | 0 |
| Nizamabad | 0.75 | 0 | 0 | Sambhal | 0.44 | 0.01 | 0 |
| East Garo Hills | 0.75 | 0 | 0 | Medak | 0.44 | 0 | 0 |
| West Jaintia Hills | 0.75 | 0 | 0 | Vaishali | 0.44 | 0 | 0 |
| South Garo Hills | 0.75 | 0 | 0 | Farrukhabad | 0.44 | 0 | 0 |
| North Goa | 0.75 | 0 | 0 | Nayagarh | 0.44 | 0 | 0 |
| Osmanabad | 0.74 | 0 | 0 | Karur | 0.44 | 0 | 0 |
| Idukki | 0.74 | 0 | 0 | Garhwa | 0.43 | 0 | 0 |
| Sultanpur | 0.74 | 0.02 | 0 | Gurgaon | 0.43 | 0 | 0 |
| South West Khasi Hills | 0.73 | 0 | 0 | Kurung Kumey | 0.43 | 0 | 0 |
| Chandauli | 0.72 | 0 | 0 | Nadia | 0.43 | 0.01 | 0.82 |
| Bangalore | 0.7 | 0.06 | 2.87 | Saharanpur | 0.43 | 0 | 0 |
| Daman | 0.7 | 0 | 0 | Batod | 0.43 | 0 | 0 |
| Mewat | 0.7 | 0 | 0 | Mysore | 0.43 | 0 | 0 |
| North East | 0.7 | 0 | 0 | Mayurbhanj | 0.43 | 0 | 0 |
| Lalitpur | 0.7 | 0 | 0 | Raichur | 0.43 | 0 | 0 |
| The Dangs | 0.69 | 0.07 | 2.46 | Saharsa | 0.43 | 0 | 0 |
| Kancheepuram | 0.69 | 0 | 0 | Purba Champaran | 0.43 | 0 | 0 |
| Ahmadabad | 0.68 | 0 | 0 | Saraikela-Kharsawan | 0.43 | 0 | 0 |
| Ghazipur | 0.67 | 0 | 0 | Jyotiba Phule Nagar | 0.43 | 0 | 0 |
| Faridkot | 0.67 | 0 | 0 | Hugli | 0.43 | 0 | 0.41 |
| Kannur | 0.66 | 0.01 | 0 | Katihar | 0.43 | 0 | 0 |
| Surguja | 0.66 | 0 | 0 | Allahabad | 0.43 | 0 | 0 |
| Bhopal | 0.64 | 0 | 0 | Koriya | 0.43 | 0 | 0 |
| Yanam | 0.63 | 0 | 0 | Kasaragod | 0.43 | 0.01 | 0 |
| Krishna | 0.63 | 0.32 | 9.43 | Kullu | 0.43 | 0 | 0 |
| Kandhamal | 0.63 | 0.01 | 0 | Nalbari | 0.43 | 0 | 0 |
| **Total (for all 652 districts in analysis)** |  | **31.27** | **87.7** |  |  | **68.73** | **12.3** |

*Total percentage share for each zone. Here, we are reporting only the top 30 districts for each zone. The full list is available upon request.

Table A4.3 Index Scores (Pakistan-Sindh)

*% of actual cases drawn from August 19, 2021*

| **Red Zone** | **CR-Index** | **% of Actual Cases** | **% of Actual Deaths** | **Green Zone** | **CR-Index** | **% of Actual Cases** | **% of Actual Deaths** |
| --- | --- | --- | --- | --- | --- | --- | --- |
| Karachi East | 0.83 | 28.94 | 32.14 | Sukkur | 0.51 | 0 | 0 |
| Karachi West | 0.73 | 1.44 | 25 | Tando Allahyar | 0.49 | 1.51 | 0 |
| Karachi Central | 0.71 | 14.13 | 3.57 | Mirpurkhas | 0.46 | 3.43 | 0 |
| Hyderabad | 0.62 | 8.3 | 0 | Umerkot | 0.45 | 0.14 | 0 |
| Karachi Malir | 0.58 | 8.02 | 0 | Jamshoro | 0.4 | 2.33 | 0 |
| Karachi South | 0.56 | 23.25 | 32.14 | Badin | 0.37 | 1.17 | 3.57 |
|  |  |  |  | Thatta | 0.35 | 0.41 | 0 |
|  |  |  |  | Larkana | 0.34 | 0 | 0 |
|  |  |  |  | S.B.A | 0.31 | 3.43 | 3.57 |
|  |  |  |  | Dadu | 0.31 | 0 | 0 |
|  |  |  |  | Sanghar | 0.31 | 0.14 | 0 |
|  |  |  |  | N. Feroze | 0.29 | 1.71 | 0 |
|  |  |  |  | Matiari | 0.29 | 1.3 | 0 |
|  |  |  |  | Kambar | 0.28 | 0 | 0 |
|  |  |  |  | T.M Khan | 0.27 | 0 | 0 |
|  |  |  |  | Ghotki | 0.26 | 0 | 0 |
|  |  |  |  | Khairpur | 0.23 | 0.14 | 0 |
|  |  |  |  | Kashmore | 0.16 | 0.07 | 0 |
|  |  |  |  | Tharparkar | 0.12 | 0.07 | 0 |
|  |  |  |  | Shikarpur | 0.11 | 0.07 | 0 |
|  |  |  |  | Jccobabad | 0.08 | 0 | 0 |
| **Total** |  | **84.09** | **92.86** |  |  | **15.91** | **7.14** |

**A5: A simple flowchart for constructing the CR-index**

Figure A5.1

Start

Decisions about constructing CR-Index for Country A

Collect Data on all 4 domains for each administrative subdivision (e.g. district)

Create sub-indices by using feature scaling (min-max) for each domain specific variable

Take arithmetic mean of the sub-indices to create the CR-index

Values for the CR-index variable represents the contagion risk for each of the administrative subdivision

End

# **B. Supplementary Time Series Figures of Covid-19 Cases and Deaths**

Figure B.1: Daily Covid-19 cases in Bangladesh


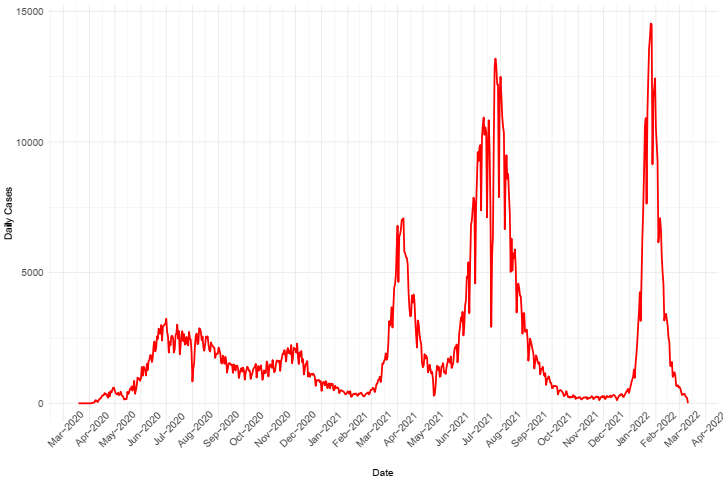


Figure B.2: Daily Covid-19 cases in India


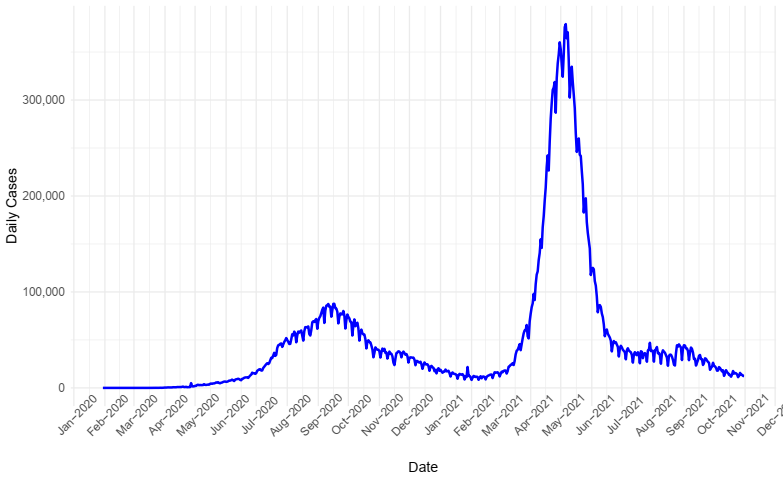


Figure B.3: Daily Covid-19 deaths in India


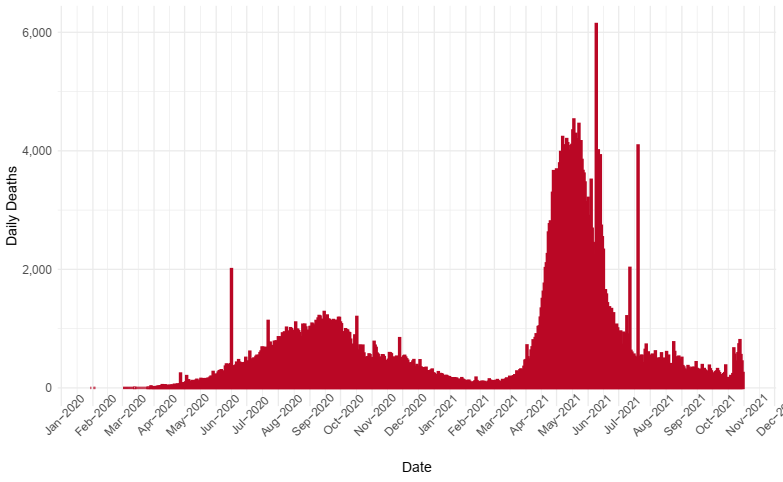


Figure B.4: Daily Covid-19 cases in Pakistan (Sindh)


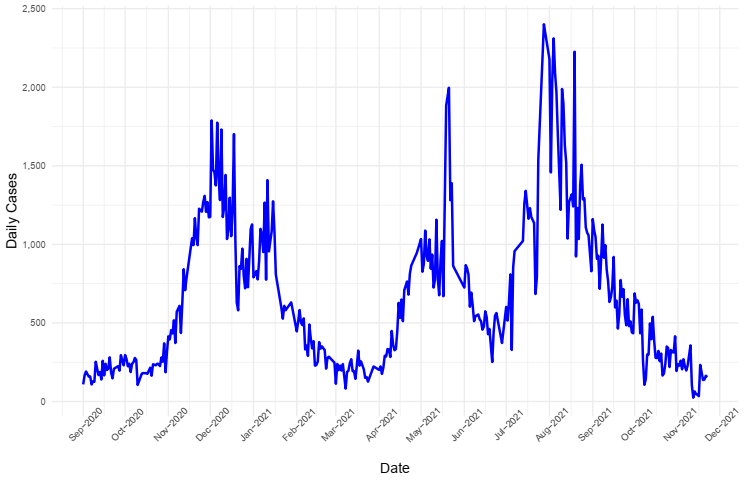


Figure B.5: Daily Covid-19 deaths in Pakistan (Sindh)


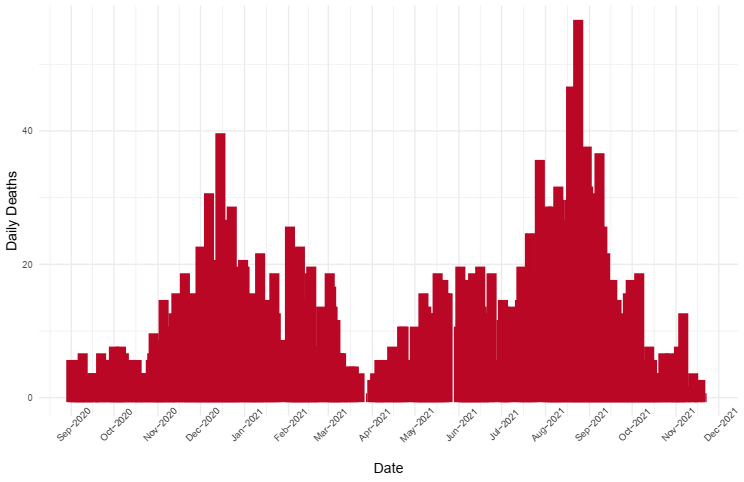


# **C. Contagion Zoning (Additional Results)**

Table C1: CR Index Percentile for Zoning

| Country | 25th percentile | Mean | 75th percentile |
| --- | --- | --- | --- |
| Bangladesh | 0.43 | 0.48 | 0.56 |
| India | 0.33 | 0.37 | 0.44 |
| Pakistan (Sindh) | 0.27 | 0.34 | 0.51 |

# **C1: Index Scores**

*District Index scores by zone and % share of actual cases by the district*

Table C1.1: Bangladesh

*% of actual cases drawn from February 10, 2022*

| **Red Zone** | **CR-Index** | **% Of Actual Cases** | **Green Zone** | **CR-Index** | **% Of Actual Cases** | **Green Zone** | **CR-Index** | **% Of Actual Cases** |
| --- | --- | --- | --- | --- | --- | --- | --- | --- |
| Chittagong | 0.69 | 6.27 | Manikganj | 0.61 | 0.86 | Pirojpur | 0.42 | 0.15 |
| Dhaka | 0.66 | 32.49 | Comilla | 0.61 | 3.89 | Lalmonirhat | 0.42 | 0.44 |
| Gazipur | 0.65 | 2.07 | Brahmanbaria | 0.61 | 1.76 | Kurigram | 0.41 | 0.22 |
| Narsingdi | 0.65 | 0.97 | Chandpur | 0.61 | 1.3 | Natore | 0.4 | 0.59 |
| Narayanganj | 0.63 | 1.65 | Tangail | 0.61 | 1.85 | Rangamati | 0.39 | 0.15 |
| Rajshahi | 0.62 | 1.28 | Feni | 0.6 | 1.23 | Patuakhali | 0.38 | 0.59 |
| Munshiganj | 0.62 | 1.19 | Jhenaidah | 0.57 | 0.4 | Jhalokati | 0.38 | 0.15 |
|  |  |  | Noakhali | 0.56 | 1.8 | Dinajpur | 0.36 | 0.99 |
|  |  |  | Rajbari | 0.56 | 0.88 | Joypurhat | 0.36 | 0.4 |
|  |  |  | Mymensingh | 0.56 | 1.3 | Gopalganj | 0.35 | 1.65 |
|  |  |  | Madaripur | 0.54 | 1.25 | Khagrachhari | 0.35 | 0.15 |
|  |  |  | Maulvibazar | 0.53 | 0.75 | Bhola | 0.32 | 0.64 |
|  |  |  | Shariatpur | 0.53 | 0.59 | Meherpur | 0.3 | 0.95 |
|  |  |  | Faridpur | 0.53 | 2.15 | Bagerhat | 0.28 | 0.37 |
|  |  |  | Thakurgaon | 0.53 | 0.2 | Bandarban | 0.27 | 0.02 |
|  |  |  | Khulna | 0.52 | 0.97 |  |  |  |
|  |  |  | Kushtia | 0.52 | 1.03 |  |  |  |
|  |  |  | Cox's Bazar | 0.51 | 4.57 |  |  |  |
|  |  |  | Jessore | 0.51 | 1.23 |  |  |  |
|  |  |  | Gaibandah | 0.51 | 0.26 |  |  |  |
|  |  |  | Kishoreganj | 0.51 | 1.49 |  |  |  |
|  |  |  | Sherpur | 0.5 | 0.18 |  |  |  |
|  |  |  | Sunamganj | 0.49 | 1.19 |  |  |  |
|  |  |  | Barguna | 0.48 | 0.15 |  |  |  |
|  |  |  | Barisal | 0.48 | 1.28 |  |  |  |
|  |  |  | Lakshmipur | 0.47 | 1.06 |  |  |  |
|  |  |  | Jamalpur | 0.47 | 0.26 |  |  |  |
|  |  |  | Nilphamari | 0.46 | 0.29 |  |  |  |
|  |  |  | Netrokona | 0.46 | 0.31 |  |  |  |
|  |  |  | Rangpur | 0.46 | 1.12 |  |  |  |
|  |  |  | Pabna | 0.46 | 2.26 |  |  |  |
|  |  |  | Magura | 0.45 | 0.26 |  |  |  |
|  |  |  | Naogaon | 0.45 | 0.75 |  |  |  |
|  |  |  | Chuadanga | 0.44 | 0.59 |  |  |  |
|  |  |  | Habiganj | 0.44 | 1.08 |  |  |  |
|  |  |  | Satkhira | 0.44 | 0.73 |  |  |  |
|  |  |  | Sylhet | 0.44 | 1.96 |  |  |  |
|  |  |  | Sirajganj | 0.44 | 0.99 |  |  |  |
|  |  |  | Chapai Nababganj | 0.44 | 0.29 |  |  |  |
|  |  |  | Panchagarh | 0.43 | 0.48 |  |  |  |
|  |  |  | Narail | 0.43 | 0.68 |  |  |  |
|  |  |  | Bogra | 0.43 | 0.97 |  |  |  |
| **Total** |  | **45.92** |  |  | **46.64** |  |  | **7.46** |

Table C1.2: India

*% of actual cases drawn from October 31, 2021*

| **Red Zone** | **CR-Index** | **% of Actual Cases** | **% of Actual Deaths** | **Orange Zone** | **CR-Index** | **% of Actual Cases** | **% of Actual Deaths** | **Green Zone** | **CR-Index** | **% of Actual Cases** | **% of Actual Deaths** |
| --- | --- | --- | --- | --- | --- | --- | --- | --- | --- | --- | --- |
| Kollam | 0.76 | 0.12 | 2.46 | Jagatsinghapur | 0.55 | 0.00 | 0.00 | Mahrajganj | 0.32 | 0.00 | 0.00 |
| Mungeli | 0.75 | 0.13 | 1.23 | Komaram Bheem | 0.55 | 0.26 | 0.00 | Malappuram | 0.32 | 0.00 | 0.00 |
| Chatra | 0.75 | 0.05 | 1.64 | Dumka | 0.55 | 0.00 | 0.00 | Chikmagalur | 0.32 | 0.00 | 0.00 |
| Sultanpur | 0.74 | 0.02 | 0.00 | Tiruvannamalai | 0.55 | 0.00 | 0.41 | Ganderbal | 0.32 | 0.00 | 0.00 |
| Chandauli | 0.72 | 0.00 | 0.00 | Amritsar | 0.55 | 0.00 | 0.00 | Jhabua | 0.32 | 0.00 | 0.00 |
| Bangalore | 0.70 | 0.06 | 2.87 | Khowai | 0.54 | 0.11 | 0.82 | Rajkot | 0.32 | 0.00 | 0.00 |
| Daman | 0.70 | 0.00 | 0.00 | Mahesana | 0.54 | 0.00 | 0.00 | Rae Bareli | 0.32 | 0.00 | 0.00 |
| Lalitpur | 0.70 | 0.00 | 0.00 | Karauli | 0.54 | 0.00 | 0.00 | Bahraich | 0.32 | 0.00 | 0.00 |
| The Dangs | 0.69 | 0.07 | 2.46 | Dindigul | 0.54 | 0.00 | 0.41 | Jashpur | 0.32 | 0.00 | 0.00 |
| Ahmadabad | 0.68 | 0.00 | 0.00 | Darjiling | 0.54 | 0.01 | 0.00 | Latehar | 0.32 | 0.00 | 0.00 |
| Ghazipur | 0.67 | 0.00 | 0.00 | Mirzapur | 0.53 | 0.00 | 0.00 | Bastar | 0.32 | 0.00 | 0.00 |
| Faridkot | 0.67 | 0.00 | 0.00 | Lohardaga | 0.53 | 0.00 | 0.00 | Basti | 0.31 | 0.00 | 0.00 |
| Kannur | 0.66 | 0.01 | 0.00 | Papum Pare | 0.53 | 0.00 | 0.00 | Barmer | 0.31 | 0.00 | 0.00 |
| Surguja | 0.66 | 0.00 | 0.00 | Vikarabad | 0.53 | 0.00 | 0.00 | Raipur | 0.31 | 0.00 | 0.00 |
| Bhopal | 0.64 | 0.00 | 0.00 | Jaisalmer | 0.52 | 0.00 | 0.00 | Tapi | 0.31 | 0.00 | 0.00 |
| Yanam | 0.63 | 0.00 | 0.00 | Palwal | 0.52 | 0.01 | 0.00 | Bid | 0.31 | 0.01 | 0.00 |
| Krishna | 0.63 | 0.32 | 9.43 | Kathua | 0.52 | 0.06 | 0.00 | Dhamtari | 0.31 | 0.00 | 0.00 |
| Kandhamal | 0.63 | 0.01 | 0.00 | Malkangiri | 0.52 | 0.14 | 19.67 | Mansa | 0.31 | 0.00 | 0.00 |
| Gwalior | 0.63 | 0.00 | 0.00 | Paschim Medinipur | 0.52 | 0.00 | 0.00 | Jalor | 0.31 | 0.00 | 0.00 |
| Tirap | 0.62 | 0.32 | 24.18 | Jamtara | 0.52 | 0.00 | 0.00 | Kanpur Nagar | 0.31 | 0.00 | 0.00 |
| Lunglei | 0.61 | 0.00 | 0.00 | Warangal (U) | 0.52 | 0.00 | 0.00 | Katni | 0.31 | 0.00 | 0.00 |
| Hapur | 0.61 | 0.03 | 0.00 | Muzaffarpur | 0.52 | 0.00 | 0.00 | South Andaman | 0.31 | 0.00 | 0.00 |
| Dhanbad | 0.61 | 0.00 | 0.00 | Thiruvallur | 0.51 | 0.01 | 0.82 | Changlang | 0.31 | 0.00 | 0.00 |
| Jabalpur | 0.61 | 0.00 | 0.00 | Rampur | 0.51 | 0.00 | 0.00 | Yavatmal | 0.31 | 0.00 | 0.00 |
| Doda | 0.60 | 0.00 | 0.00 | Ambala | 0.51 | 0.00 | 0.00 | Bijapur | 0.31 | 0.00 | 0.00 |
| Ludhiana | 0.60 | 0.00 | 0.00 | Coimbatore | 0.51 | 0.05 | 0.41 | Bulandshahr | 0.31 | 0.00 | 0.00 |
| Erode | 0.60 | 0.45 | 4.51 | Karimnagar | 0.51 | 0.00 | 0.00 | Palakkad | 0.31 | 0.01 | 0.00 |
| Nuapada | 0.60 | 0.06 | 2.46 | Agra | 0.51 | 0.00 | 0.00 | Kendrapara | 0.31 | 0.00 | 0.00 |
| Thiruvarur | 0.59 | 0.01 | 0.00 | Kozhikode | 0.50 | 0.22 | 2.05 | Shupiyan | 0.31 | 0.00 | 0.00 |
| Kanpur Dehat | 0.59 | 0.13 | 0.00 | South West Garo Hills | 0.50 | 0.03 | 0.41 | Khammam | 0.31 | 0.00 | 0.00 |
| **Total (for all 652 districts in analysis)** |  | 2.65 | 52.87 |  |  | 97.26 | 47.13 |  |  | 0.09 | 0.00 |

*Total percentage share for each zone. Here, we are reporting only the top 30 districts for each zone. The full list is available upon request.

Table C1.3 Pakistan (Sindh)

*% of actual cases drawn from August 19, 2021*

| **Red Zone** | **CR-Index** | **% of Actual Cases** | **% of Actual Deaths** | **Orange Zone** | **CR-Index** | **% of Actual Cases** | **% of Actual Deaths** | **Green Zone** | **CR-Index** | **% of Actual Cases** | **% of Actual Deaths** |
| --- | --- | --- | --- | --- | --- | --- | --- | --- | --- | --- | --- |
| Karachi East | 0.83 | 28.94 | 32.14 | Hyderabad | 0.62 | 8.3 | 0 | T.M Khan | 0.27 | 0 | 0 |
| Karachi West | 0.73 | 1.44 | 25 | Karachi Malir | 0.58 | 8.02 | 0 | Ghotki | 0.26 | 0 | 0 |
| Karachi Central | 0.71 | 14.13 | 3.57 | Karachi South | 0.56 | 23.25 | 32.14 | Khairpur | 0.23 | 0.14 | 0 |
|  |  |  |  | Sukkur | 0.51 | 0 | 0 | Kashmore | 0.16 | 0.07 | 0 |
|  |  |  |  | Tando Allahyar | 0.49 | 1.51 | 0 | Tharparkar | 0.12 | 0.07 | 0 |
|  |  |  |  | Mirpurkhas | 0.46 | 3.43 | 0 | Shikarpur | 0.11 | 0.07 | 0 |
|  |  |  |  | Umerkot | 0.45 | 0.14 | 0 | Jccobabad | 0.08 | 0 | 0 |
|  |  |  |  | Jamshoro | 0.4 | 2.33 | 0 |  |  |  |  |
|  |  |  |  | Badin | 0.37 | 1.17 | 3.57 |  |  |  |  |
|  |  |  |  | Thatta | 0.35 | 0.41 | 0 |  |  |  |  |
|  |  |  |  | Larkana | 0.34 | 0 | 0 |  |  |  |  |
|  |  |  |  | S.B.A | 0.31 | 3.43 | 3.57 |  |  |  |  |
|  |  |  |  | Dadu | 0.31 | 0 | 0 |  |  |  |  |
|  |  |  |  | Sanghar | 0.31 | 0.14 | 0 |  |  |  |  |
|  |  |  |  | N. Feroze | 0.29 | 1.71 | 0 |  |  |  |  |
|  |  |  |  | Matiari | 0.29 | 1.3 | 0 |  |  |  |  |
|  |  |  |  | Kambar | 0.28 | 0 | 0 |  |  |  |  |
| **Total** |  | **44.51** | **60.71** |  |  | **55.14** | **39.28** |  |  | **0.35** | **0** |

# **D. Tables on Predictive Performance for the CR-Index**

We use multiclass classification to assign districts into three risk groups based on three colored zones. For a given country, a district is identified as high-risk and ‘Red’ zone, if the percentage of COVID-19 cases (or deaths) in that district exceeds the 90^th^ percentile threshold relative to all other districts in the country. A district is identified as ‘Orange’ zone if the percentage of COVID-19 cases (or deaths) in that district is between the 90^th^ and 25^th^ percentile relative to other districts; and ‘Green’ zone if below the 25^th^ percentile. In our multiclass classification, positive class is defined by districts that are identified as high-risk or ‘Red’ zones. We then model the multiclass categorical response variable for district-wise COVID -19 cases (deaths) as a function of the CR-Index for the training data to feed the RF algorithm. We then compare the predicted risk category of each district, with their actual risk category in the test data. We use the train/test split method where the RF process is fitted to the training data, and its predictive accuracy is assessed with the test sample. In contrast to the traditional approach of using a random split to determine the test data, we use the early/late split method, where the early time-series data is utilized to train, and the later data is used as a test. Moreover, to reduce the bias and variance of the estimations, we fit RF using 5-fold cross validation repeated for 50 times.

The classification prediction of the CR-Index is assessed by the AUC (Area Under the Curve) and ROC (Receiver Operating Characteristics) curves. ROC is a probability curve that informs how much the CR-Index can distinguish between classes, whereas AUC measures the degree of separability. The ROC curve plots the true positive rate (TPR) against the false positive rate (FPR). TPR, also known as “Sensitivity”, is the ratio of districts that are correctly categorized as high-risk (true positive) to the total number of positives. Whereas FPR, is “(1 – Specificity)”, where “Specificity” is the true negative rate, and it is the ratio of the number of low-risk districts incorrectly categorized as high-risk to the total number of actual negatives. Each point on the ROC curves represents a specific sensitivity-specificity pair corresponding to a particular decision threshold.

Higher the value of AUC – greater the predictive accuracy – and therefore, the better the model (here the CR-Index) at distinguishing high-risk districts from low-risk. For example, when AUC equals the value of one, the ROC curve reaches the upper top-left corner of the plot implying 100% test accuracy, or perfect separation between the two risk categories.

In general, an AUC value between 0.70 to 0.80 is considered acceptable; 0.80 to 0.90 is considered excellent, and more than 0.90 is considered outstanding

In addition to AUC, the detailed tables also report the values of other commonly used metrics for predictive performance, namely, sensitivity, specificity, and balanced accuracy. Sensitivity is defined as the true positive rate, whereas specificity is the true negative rate. High-risk (Red) districts are taken as the positive class. Balanced accuracy is the arithmetic average of sensitivity and specificity.

# **D1: Tables on Predictive Performance for the CR-Index (Multi classification)**

Table D1.1: 2020 data as training and 2021 as test using multi-classification (India Covid-19 cases)

| **Month** | **Balanced Accuracy** | **Sensitivity** | **Specificity** | **AUC-ROC** |
| --- | --- | --- | --- | --- |
| January | 0.74 | 0.68 | 0.80 | 0.79 |
| February | 0.66 | 0.58 | 0.74 | 0.71 |
| March | 0.65 | 0.56 | 0.74 | 0.71 |
| April | 0.78 | 0.72 | 0.84 | 0.82 |
| May | 0.85 | 0.81 | 0.89 | 0.88 |
| June | 0.66 | 0.57 | 0.74 | 0.72 |
| July | 0.60 | 0.52 | 0.67 | 0.66 |
| August | 0.59 | 0.52 | 0.66 | 0.65 |
| September | 0.62 | 0.56 | 0.68 | 0.68 |
| October | 0.62 | 0.55 | 0.69 | 0.67 |
| **Mean** | **0.68** | **0.61** | **0.75** | **0.73** |
| **2021** | **0.86** | **0.83** | **0.89** | **0.90** |
| The table provides the predictive accuracy results using multi-classification. Districts belong to the red zone if covid-19 cases are greater than or equal to the 90th percentile; orange zone if cases fall within the 90th and 25th percentile; and green zone if below the 25th percentile. The mean computes the average of balanced accuracy, sensitivity, specificity, and AUC-ROC values across all the available months in 2021 used separately as testing data and 2020 as the training data. The values for the 2021 row use the entire 2021 as the testing data and 2020 as the training using the early-late split method of cross validation. | | | | |

Table D1.2: 2020 data as training and 2021 as test using multi-classification (India Covid-19 deaths)

| **Month** | **Balanced Accuracy** | **Sensitivity** | **Specificity** | **AUC-ROC** |
| --- | --- | --- | --- | --- |
| January | 0.68 | 0.59 | 0.78 | 0.73 |
| February | 0.75 | 0.55 | 0.95 | 0.84 |
| March | 0.75 | 0.54 | 0.95 | 0.79 |
| April | 0.73 | 0.64 | 0.81 | 0.77 |
| May | 0.75 | 0.68 | 0.82 | 0.81 |
| June | 0.72 | 0.64 | 0.80 | 0.78 |
| July | 0.61 | 0.51 | 0.71 | 0.69 |
| August | 0.68 | 0.42 | 0.94 | 0.71 |
| September | 0.74 | 0.55 | 0.93 | 0.79 |
| October | 0.71 | 0.51 | 0.91 | 0.78 |
| **Mean** | **0.71** | **0.56** | **0.86** | **0.77** |
| **2021** | **0.86** | **0.83** | **0.89** | **0.84** |
| The table provides the predictive accuracy results using multi-classification. Districts belong to the red zone if covid-19 deaths are greater than or equal to the 90th percentile; orange zone if deaths fall within the 90th and 25th percentile; and green zone if below the 25th percentile. The mean computes the average of balanced accuracy, sensitivity, specificity, and AUC-ROC values across all the available months in 2021 used separately as testing data and 2020 as the training data. The values for the 2021 row use the entire 2021 as the testing data and 2020 as the training using the early-late split method of cross validation. | | | | |

Table D1.3: 2020 data as training and 2021 as test using multi-classification (Pakistan-Sindh Covid-19 cases)

| **Month** | **Balanced Accuracy** | **Sensitivity** | **Specificity** | **AUC-ROC** |
| --- | --- | --- | --- | --- |
| January | 0.82 | 0.79 | 0.84 | 0.87 |
| February | 0.62 | 0.54 | 0.71 | 0.82 |
| March | 0.62 | 0.54 | 0.71 | 0.82 |
| April | 0.72 | 0.70 | 0.75 | 0.92 |
| May | 0.75 | 0.72 | 0.79 | 0.93 |
| July | 0.82 | 0.79 | 0.84 | 0.87 |
| August | 0.78 | 0.74 | 0.82 | 0.86 |
| October | 0.72 | 0.70 | 0.75 | 0.85 |
| **Mean** | **0.73** | **0.69** | **0.78** | **0.87** |
| **2021** | **0.84** | **0.83** | **0.85** | **0.97** |
| The table provides the predictive accuracy results using multi-classification. Districts belong to the red zone if covid-19 cases are greater than or equal to the 90th percentile; orange zone if cases fall within the 90th and 25th percentile; and green zone if below the 25th percentile. The mean computes the average of balanced accuracy, sensitivity, specificity, and AUC-ROC values across all the available months in 2021 used separately as testing data and 2020 as the training data. The values for the 2021 row use the entire 2021 as the testing data and 2020 as the training using the early-late split method of cross validation. The predictive accuracy for the month of June is missing due to computational reasons. | | | | |

Table D1.4: 2020 data as training and 2021 as test using multi-classification (Pakistan-Sindh Covid-19 deaths)

| **Month** | **Balanced Accuracy** | **Sensitivity** | **Specificity** | **AUC-ROC** |
| --- | --- | --- | --- | --- |
| January | 0.71 | 0.68 | 0.75 | 0.77 |
| February | 0.72 | 0.68 | 0.76 | 0.73 |
| March | 1.00 | 1.00 | 1.00 | 1.00 |
| April | 0.81 | 0.67 | 0.96 | 0.94 |
| May | 0.81 | 0.69 | 0.82 | 0.91 |
| June | 0.76 | 0.73 | 0.80 | 0.86 |
| July | 0.65 | 0.56 | 0.75 | 0.72 |
| August | 0.79 | 0.72 | 0.86 | 0.92 |
| September | 0.80 | 0.76 | 0.83 | 0.93 |
| October | 0.61 | 0.51 | 0.72 | 0.63 |
| **Mean** | **0.77** | **0.70** | **0.83** | **0.84** |
| **2021** | **0.91** | **0.90** | **0.91** | **0.96** |
| The table provides the predictive accuracy results using multi-classification. Districts belong to the red zone if covid-19 deaths are greater than or equal to the 90th percentile; orange zone if deaths fall within the 90th and 25th percentile; and green zone if below the 25th percentile. The mean computes the average of balanced accuracy, sensitivity, specificity, and AUC-ROC values across all the available months in 2021 used separately as testing data and 2020 as the training data. The values for the 2021 row use the entire 2021 as the testing data and 2020 as the training using the early-late split method of cross validation. | | | | |

Table D1.5: 2020 data as training and 2021 as test using multi-classification (Bangladesh Covid-19 cases)

| **Month** | **Balanced Accuracy** | **Sensitivity** | **Specificity** | **AUC-ROC** |
| --- | --- | --- | --- | --- |
| January | 0.78 | 0.75 | 0.80 | 0.82 |
| February | 0.70 | 0.65 | 0.76 | 0.77 |
| March | 0.68 | 0.62 | 0.74 | 0.76 |
| April | 0.68 | 0.62 | 0.74 | 0.77 |
| May | 0.66 | 0.58 | 0.77 | 0.71 |
| June | 0.63 | 0.52 | 0.74 | 0.67 |
| July | 0.73 | 0.66 | 0.81 | 0.80 |
| August | 0.66 | 0.56 | 0.77 | 0.73 |
| September | 0.67 | 0.58 | 0.77 | 0.78 |
| October | 0.67 | 0.57 | 0.77 | 0.72 |
| November | 0.70 | 0.61 | 0.80 | 0.74 |
| December | 0.68 | 0.65 | 0.70 | 0.78 |
| January | 0.72 | 0.67 | 0.77 | 0.82 |
| February | 0.74 | 0.66 | 0.82 | 0.78 |
| **Mean** | **0.69** | **0.62** | **0.77** | **0.76** |
| **2021** | **0.78** | **0.74** | **0.83** | **0.86** |
| **2022** | **0.72** | **0.65** | **0.79** | **0.79** |
| The table provides the predictive accuracy results using multi-classification. Districts belong to the red zone if covid-19 cases are greater than or equal to the 90th percentile; orange zone if cases fall within the 90th and 25th percentile; and green zone if below the 25th percentile. The mean computes the average of balanced accuracy, sensitivity, specificity, and AUC-ROC values across all the available months in 2021 used separately as testing data and 2020 as the training data. The values for the 2021 row use the entire 2021 as the testing data and 2020 as the training using the early-late split method of cross validation. | | | | |

# **D2: Tables on Predictive Performance for the CR-Index (Binary Classification using 75^th^ percentile cut-off)**

Table D2.1: 2020 data as training and 2021 as test using 0.75 percentile cut-off (India Covid-19 cases)

| **Month** | **Balanced Accuracy** | **Sensitivity** | **Specificity** | **AUC-ROC** |
| --- | --- | --- | --- | --- |
| January | 0.84 | 0.85 | 0.82 | 0.84 |
| February | 0.84 | 0.94 | 0.74 | 0.86 |
| March | 0.81 | 0.86 | 0.77 | 0.83 |
| April | 0.84 | 0.77 | 0.91 | 0.85 |
| May | 0.88 | 0.80 | 0.97 | 0.90 |
| June | 0.82 | 0.78 | 0.87 | 0.84 |
| July | 0.80 | 0.82 | 0.78 | 0.82 |
| August | 0.80 | 0.86 | 0.74 | 0.81 |
| September | 0.77 | 0.84 | 0.71 | 0.78 |
| October | 0.77 | 0.82 | 0.73 | 0.77 |
| **Mean** | **0.82** | **0.83** | **0.80** | **0.83** |
| **2021** | **0.91** | **0.87** | **0.96** | **0.92** |
| The mean computes the average of balanced accuracy, sensitivity, specificity, and AUC-ROC values across all the available months in 2021 used separately as testing data and 2020 as the training data. The values for the 2021 row use the entire 2021 as the testing data and 2020 as the training using the early-late split method of cross validation. | | | | |

Table D2.2: 2020 data as training and 2021 as test using 0.75 percentile cut-off (Indian Covid-19 deaths)

| **Month** | **Balanced Accuracy** | **Sensitivity** | **Specificity** | **AUC-ROC** |
| --- | --- | --- | --- | --- |
| January | 0.79 | 0.71 | 0.87 | 0.81 |
| February | 0.80 | 0.77 | 0.83 | 0.82 |
| March | 0.79 | 0.76 | 0.82 | 0.82 |
| April | 0.78 | 0.66 | 0.90 | 0.80 |
| May | 0.80 | 0.65 | 0.96 | 0.85 |
| June | 0.80 | 0.69 | 0.90 | 0.81 |
| July | 0.77 | 0.72 | 0.82 | 0.77 |
| August | 0.77 | 0.75 | 0.80 | 0.79 |
| September | 0.76 | 0.76 | 0.75 | 0.78 |
| October | 0.76 | 0.80 | 0.72 | 0.78 |
| **Mean** | **0.78** | **0.73** | **0.84** | **0.80** |
| **2021** | **0.85** | **0.74** | **0.96** | **0.88** |
| The mean computes the average of balanced accuracy, sensitivity, specificity, and AUC-ROC values across all the available months in 2021 used separately as testing data and 2020 as the training data. The values for the 2021 row use the entire 2021 as the testing data and 2020 as the training using the early-late split method of cross validation. | | | | |

Table D2.3: 2020 data as training and 2021 as test using 0.75 percentile cut-off (Pakistan-Sindh Covid-19 cases)

| **Month** | **Balanced Accuracy** | **Sensitivity** | **Specificity** | **AUC-ROC** |
| --- | --- | --- | --- | --- |
| January | 0.90 | 0.86 | 0.95 | 0.90 |
| February | 0.88 | 0.75 | 1.00 | 0.86 |
| March | 0.79 | 0.63 | 0.95 | 0.76 |
| April | 0.79 | 0.63 | 0.95 | 0.82 |
| May | 0.79 | 0.63 | 0.95 | 0.82 |
| June | 0.88 | 0.75 | 1.00 | 0.85 |
| July | 0.88 | 0.75 | 1.00 | 0.86 |
| August | 0.88 | 0.75 | 1.00 | 0.86 |
| September | 0.75 | 0.56 | 0.94 | 0.69 |
| October | 0.61 | 0.38 | 0.84 | 0.58 |
| **Mean** | **0.82** | **0.67** | **0.96** | **0.80** |
| **2021** | **0.88** | **0.75** | **1.00** | **0.86** |
| The mean computes the average of balanced accuracy, sensitivity, specificity, and AUC-ROC values across all the available months in 2021 used separately as testing data and 2020 as the training data. The values for the 2021 row use the entire 2021 as the testing data and 2020 as the training using the early-late split method of cross validation. | | | | |

Table D2.4: 2020 data as training and 2021 as test using 0.75 percentile cut-off (Pakistan-Sindh Covid-19 deaths)

| **Month** | **Balanced Accuracy** | **Sensitivity** | **Specificity** | **AUC-ROC** |
| --- | --- | --- | --- | --- |
| January | 0.90 | 0.86 | 0.95 | 0.99 |
| February | 0.83 | 0.67 | 1.00 | 0.86 |
| March | 0.93 | 1.00 | 0.86 | 1.00 |
| April | 0.83 | 0.67 | 1.00 | 0.93 |
| May | 0.88 | 0.75 | 1.00 | 0.85 |
| June | 0.88 | 0.75 | 1.00 | 0.93 |
| July | 0.81 | 0.67 | 0.94 | 0.84 |
| August | 0.88 | 0.75 | 1.00 | 0.86 |
| September | 0.83 | 0.67 | 1.00 | 0.85 |
| October | 0.75 | 0.50 | 1.00 | 0.76 |
| **Mean** | **0.85** | **0.73** | **0.98** | **0.89** |
| **2021** | **0.90** | **0.86** | **0.95** | **0.90** |
| The mean computes the average of balanced accuracy, sensitivity, specificity, and AUC-ROC values across all the available months in 2021 used separately as testing data and 2020 as the training data. The values for the 2021 row use the entire 2021 as the testing data and 2020 as the training using the early-late split method of cross validation. | | | | |

Table D2.5: 2020 data as training and 2021 as test using 0.75 percentile cut-off (Bangladesh Covid-19 cases)

| **Month** | **Balanced Accuracy** | **Sensitivity** | **Specificity** | **AUC-ROC** |
| --- | --- | --- | --- | --- |
| January | 0.87 | 0.92 | 0.83 | 0.85 |
| February | 0.84 | 0.85 | 0.84 | 0.91 |
| March | 0.85 | 0.91 | 0.79 | 0.92 |
| April | 0.91 | 0.93 | 0.90 | 0.91 |
| May | 0.85 | 0.72 | 0.98 | 0.82 |
| June | 0.77 | 0.58 | 0.96 | 0.75 |
| July | 0.84 | 0.68 | 1.00 | 0.85 |
| August | 0.78 | 0.65 | 0.91 | 0.90 |
| September | 0.82 | 0.71 | 0.94 | 0.86 |
| October | 0.83 | 0.75 | 0.92 | 0.88 |
| November | 0.88 | 0.81 | 0.94 | 0.89 |
| December | 0.83 | 0.85 | 0.82 | 0.85 |
| January | 0.87 | 0.86 | 0.88 | 0.92 |
| February | 0.82 | 0.71 | 0.94 | 0.84 |
| **Mean** | **0.84** | **0.78** | **0.90** | **0.87** |
| **2021** | **0.90** | **0.82** | **0.98** | **0.91** |
| **2022** | **0.85** | **0.80** | **0.90** | **0.87** |
| The mean computes the average of balanced accuracy, sensitivity, specificity, and AUC-ROC values across all the available months in 2021 and 2022 used separately as testing data and 2020 as the training data. The values for the 2021 and 2022 row uses the entire 2021 and 2022 as the testing data and 2020 as the training using the early-late split method of cross validation. | | | | |

# **D3: Tables on Predictive Performance for the CR-Index (District-Monthly Level)**

Table D3.1: Predictive Performance of the CR-Index using district-monthly level data

|  | India (cases) | | India (deaths) | | Pakistan-Sindh (cases) | | Pakistan-Sindh (deaths) | | Bangladesh | |
| --- | --- | --- | --- | --- | --- | --- | --- | --- | --- | --- |
|  | 75th | Multi (90) | 75th | Multi (90) | 75th | Multi (90) | 75th | Multi (90) | 75th | Multi (90) |
| Sensitivity | 0.70 | 0.53 | 0.51 | 0.53 | 0.70 | 0.66 | 0.83 | 0.70 | 0.63 | 0.58 |
| Specificity | 0.86 | 0.73 | 0.91 | 0.78 | 0.95 | 0.75 | 0.94 | 1.00 | 0.94 | 0.80 |
| Balanced Accuracy | 0.78 | 0.63 | 0.71 | 0.65 | 0.82 | 0.70 | 0.89 | 0.85 | 0.79 | 0.69 |
| AUC-ROC | 0.87 | 0.77 | 0.83 | 0.77 | 0.85 | 0.78 | 0.92 | 0.98 | 0.88 | 0.83 |

Figure D1: ROC Curves

Figure D1 provides the ROC curves for the binary classification problem, where districts are classified as high-risk if Covid-19 cases (or deaths) exceed the 75^th^ percentile threshold and vice-versa. The ROC curves are obtained by using the data for 2021 as test and 2020 as training.


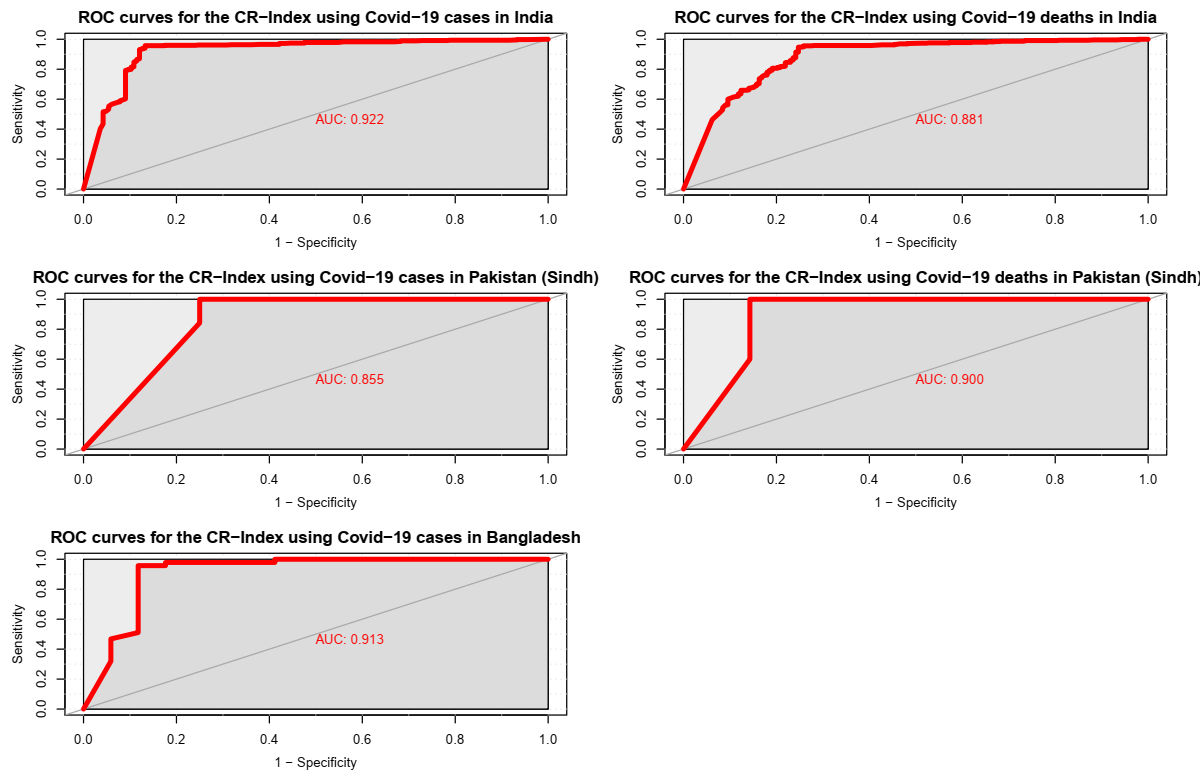

Supplement: Supplementary file 1 — Supplementary Information. [file 41598_2023_30348_MOESM1_ESM.docx]
